# A Technology-Driven Assistive Learning Tool and Framework for Personalized Dyscalculia Interventions

**DOI:** 10.3390/ejihpe15050085

**Published:** 2025-05-15

**Authors:** Dipti Jadhav, Sarat Kumar Chettri, Amiya Kumar Tripathy, Manob Jyoti Saikia

**Affiliations:** 1 Biomedical Sensors & Systems Lab, University of Memphis, Memphis, TN 38152, USA; 2 Department of Computer Engineering, Don Bosco Institute of Technology, Mumbai 400070, India; 3 Department of Computer Applications, Assam Don Bosco University, Guwahati 781017, India; 4 Electrical and Computer Engineering Department, University of Memphis, Memphis, TN 38152, USA

**Keywords:** dynamic Bayesian networks, dyscalculia, empirical research, learning disabilities, mixed methods research, self-directed learning

## Abstract

Recognizing the impact of mathematical learning difficulties on student achievement, this research focuses on developing adaptive, technology-based solutions for those struggling with learning mathematics, including individuals with dyscalculia. Dyscalculia, a difficulty in understanding numbers and mathematics, can profoundly affect a child’s academic progress and self-confidence. Many interventions aim for broad effectiveness but often struggle to address individual learning differences. This research addresses this gap by employing Dynamic Bayesian Networks (DBNs) within intelligent tutoring systems to develop a personalized, gamified approach for improving mathematical skills in children with dyscalculia. We assessed 158 children aged 6–10 years using the Smartick Dyscalculia Assessment Tool to identify specific numerical cognition deficits. Based on these assessments, we have developed EDSense (Early Detection and Intervention for Insufficient Number Sense), an adaptive web-based learning tool. EDSense provides personalized support and targets skill refinement in mathematics learning. A pre-test and post-test design evaluates EDSense’s effectiveness and demonstrates significant improvements in numerical abilities. The findings highlight the crucial role of adaptive learning platforms in addressing dyscalculia. The EDSense platform demonstrates gamified, self-directed learning environments to enhance both engagement and learning outcomes by accommodating individual cognitive differences. We have proposed a technology-driven framework for personalized dyscalculia interventions, emphasizing early detection to support mathematical skill development.

## 1. Introduction

This research focuses on methods to improve cognitive and academic abilities. However, most approaches, such as educational programs, tutoring systems, skill-refinement therapies, gamified learning strategies, and adaptive technologies, only assess overall effectiveness. They often overlook individual response variations and fail to perform in-depth analysis of key factors. In the context of learning disabilities (LDs), which are neurological diseases that impair the brain’s ability to process information and cause problems with language, coordination, and attention, the oversight of not considering individual response variations and underlying factors is significant. One such disorder that affects children’s numerical cognition and poses serious difficulties to their arithmetic ability is dyscalculia, a mathematical disorder (First, 2010; Fletcher et al., 2018).

Children with dyscalculia often struggle with number recognition, associating numeric symbols with their verbal counterparts, and discerning patterns or sequences. Beyond the classroom, these difficulties extend to daily tasks such as remembering postal codes, handling financial matters, distinguishing left from right, and reading time (Koumoula et al., 2004). Developmental dyscalculia (DD) is a specific learning disability that arises naturally during childhood and affects the ability to acquire basic numerical and arithmetic skills despite adequate intelligence and education. The prevalence of DD is estimated to affect around 3% to 6% of school-age children and is frequently associated with conditions such as Attention Deficit Hyperactivity Disorder (ADHD) and dyslexia (Koumoula et al., 2004; R. Shalev, 2004; R. S. Shalev et al., 2000). In fact, about 40% of people with dyslexia also experience difficulties with mathematics (Lewis et al., 1994).

Special education programs aim to provide personalized support to students with LDs within the general education system, optimizing their chances of success (Yell & Drasgow, 2007). Intelligent tutoring systems have shown promise in various educational areas, including providing support to students with LDs. These systems personalize the learning process by adapting the difficulty level based on estimates and predictions of the student’s knowledge (Woolf, 2010). However, the effectiveness of ITS depends heavily on accurate modeling and prediction of student knowledge, which is based on the properly calibrated parameters of the probabilistic models used (R. S. Baker et al., 2004; Corbett & Anderson, 1994).

A Dynamic Bayesian Network (DBN) is a type of probabilistic graphical model that is used to represent systems that evolve over time. It extends a Bayesian network (BN) by introducing a time dimension, allowing the modeling of how variables change and influence each other in different time steps.

In the context of student modeling, DBNs are particularly useful for tracking the evolution of learner knowledge, skills, and behaviors over time. Each time slice in a DBN represents a learning moment (such as answering a question or engaging with a task) and includes variables like the student’s knowledge state, performance, or engagement level. The connections within a time slice model how these variables interact, and the connections between slices model the progression of the learner over time (Käser et al., 2017).

Despite the potential benefits of intelligent tutoring systems, it remains challenging to effectively address the unique needs of children with dyscalculia. This research focuses on addressing these challenges by leveraging DBNs within intelligent tutoring systems and self-directed learning to address the arithmetic difficulties and knowledge structure issues faced by dyscalculic children. The primary objectives of this research include the following:

- Use DBNs for knowledge structuring within intelligent tutoring systems to improve arithmetic comprehension and performance in children with dyscalculia.
- Use gamification to improve learning outcomes and engagement.
- Ensure accurate evaluations through skill-refinement therapies using the Smartick Dyscalculia Assessment Tool, which was administered to 158 children between the ages of 6 and 10.
- Develop and test a web-based adaptive learning tool called Early Detection and Intervention for Insufficient Number Sense (EDSense).

EDSense aims to improve the mathematical skills of primary school children by addressing learning challenges and offering effective support for diverse learners. This research emphasizes the importance of targeted interventions and innovative methods to address the specific needs of children with dyscalculia. Personalized intervention and advanced techniques contribute to a deeper understanding of personalized approaches in educational interventions.

In this context, the core challenges associated with dyscalculia include difficulty with number recognition, understanding numerical concepts, performing arithmetic operations, and maintaining mathematical memory. These challenges hinder the child’s ability to grasp fundamental arithmetic skills, which are further intensified by the lack of flexible and individualized learning resources. There is a significant gap in the availability of effective therapies for students with dyscalculia, both globally and specifically in areas with limited support for learning disabilities. The unique cognitive characteristics of dyscalculic students are often not taken into account by traditional teaching approaches, which results in ongoing problems and a growing achievement gap. In order to close this gap and improve the academic performance and overall learning experience of these learners, our research will provide a novel data-driven strategy (American Psychiatric Association, 2013; Anderson, 2020; De Smedt & Gilmore, 2011).

In conclusion, by demonstrating the efficacy of cutting-edge adaptive learning tools and approaches, this research aims to significantly improve the field of educational interventions for students with dyscalculia. The research highlights the important need for specialized educational support that can help children with dyscalculia reach their full potential in mathematics and beyond by emphasizing individualized approaches. This research makes several important contributions to the field of educational interventions for children with dyscalculia:

- Innovative use of technology—This showcases the effective use of DBNs to create personalized learning experiences, tailoring interventions to meet the unique needs of each child.
- Engagement through gamification—The research introduces a gamified learning approach that makes mathematics more engaging and enjoyable for students with dyscalculia, helping to motivate them and improve their learning outcomes.
- Accurate skill assessment—By employing the Smartick Dyscalculia Assessment Tool, the research provides a reliable method to evaluate children’s arithmetic skills and identify areas needing improvement.
- Development of EDSense—The EDSense tool offers a practical solution for the early identification and support of children struggling with mathematics.
- Focus on individualized support—This research emphasizes the importance of personalized educational support, with the aim of helping children with dyscalculia realize their full potential in mathematics and other areas of learning.

## 2. Literature Review

In recent years, the integration of artificial intelligence (AI) and digital tools in primary and early childhood education has received significant academic attention. Aravantinos et al. (2024) conducted a systematic review of educational approaches using AI in primary school settings. They identified both opportunities and pedagogical challenges. Their findings emphasize the transformative potential of AI-powered educational tools, particularly to foster personalized learning and enhance student engagement. However, they also note a critical need for teacher training and curriculum redesign to fully leverage the benefits of AI. AI offers new opportunities for personalized learning; its application could also support students with specific learning difficulties, such as dyscalculia, by addressing their unique cognitive and educational needs.

Severe difficulties in learning arithmetic are common among children, with dyscalculia just one of several reasons for such challenges. Despite varied definitions, there is agreement on the behavioral characteristics associated with dyscalculia, including an inability to quickly perceive small quantities without counting (subitizing), which reflects a core deficit in the number sense. Other manifestations of poor number sense include difficulties with estimation, counting backward reliably, comparing numbers, and confusion with mathematical signs. Dyscalculic children can also rely on inefficient counting strategies with fingers instead of using more efficient calculation methods. Beyond number sense, children with dyscalculia often face challenges in money management and aspects of time, such as reading analog clocks and managing time in daily life. In addition, they may struggle with memory, experiencing problems with both short-term and long-term recall, such as difficulties in learning and retaining multiplication tables, as well as challenges with sequencing and understanding directional concepts such as left and right or east and west (Williams, 2012).

Given these multifaceted difficulties, the use of fingers could serve as a valuable and complementary tool in addressing these challenges. Integrating finger use into early mathematics education could help children develop more effective mental number representations, supporting their learning of counting and calculation. More research is needed to investigate whether explicitly incorporating finger use could benefit children without developmental disorders and whether this approach could facilitate the shift from concrete to abstract number representations for both children with and without dyscalculia (Kaufmann, 2008).

Research has suggested that tools such as Smartick can provide a comprehensive and efficient approach to assessing dyscalculia (Smartick Method, 2022). The adaptive algorithms used by Smartick can adjust the difficulty level of the exercises based on the individual’s performance, providing a customized assessment experience that is aligned with the student’s mathematical learning abilities.

Adaptive learning, also known as adaptive teaching, involves recommending personalized learning items (e.g., lectures and exercises) in a sequential manner to meet the unique needs of each learner. Existing methods often focus separately on the knowledge levels of learners or the knowledge structure of the learning elements, despite the importance of modeling the cognitive structure, which includes both knowledge levels and knowledge structure, for optimal learning path recommendation (Liu et al., 2019).

Optimizing the learning process by offering an optimal level of cognitive stimulation through topological models can enhance active engagement, motivation, and cognitive development in students. This can involve adjusting the complexity or difficulty of the topological models to match the cognitive abilities and learning styles of students, providing an optimal level of challenge that promotes learning and skill development.

Several studies have explored the application of Bayesian networks in modeling skill topologies to better understand the knowledge structure and dynamics of learning progressions. Bayesian networks are utilized to model skill dependencies in a mathematics domain, unveiling the sequential relationships among skills and their dependencies as a Directed Acyclic Graph (DAG). Bayesian networks are found to effectively capture the hierarchical structure of skills and their probabilistic dependencies, providing insights into the typical order in which skills are learned (R. S. J. D. Baker & Yacef, 2009).

Applied Bayesian networks are utilized to model the skill topology in a programming domain, enabling the identification of prerequisite relationships among skills and predicting student performance. The findings demonstrated the effectiveness of Bayesian networks in capturing complex relationships among skills and predicting student performance with high accuracy (De La Vara & García-Serrano, 2014).

Bayesian networks can be utilized to model skill topologies in a language learning context, revealing the interdependencies among language skills and their influence on language proficiency. Research has shown that Bayesian networks can offer valuable information on the structure of language learning and help identify critical skills that have a significant impact on language proficiency (Kim et al., 2017).

Recent studies highlight the potential of Bayesian networks in modeling skill topologies and their dependencies, enabling a deeper understanding of the structure and dynamics of learning progressions. Bayesian networks provide a flexible and probabilistic method for capturing intricate relationships between skills, making them a powerful tool in educational data mining and learning analytics research.

The methodology for constructing Bayesian networks to model skill topologies is presented in this article. It involves defining the skills, specifying the dependencies between the skills, and estimating the parameters of the Bayesian network from student performance data. The constructed Bayesian networks can be used for various tasks, such as predicting students’ performance in unobserved skills, diagnosing skill deficiencies, and generating personalized recommendations for remedial actions. This approach provides a probabilistic graphical model that captures the relationships between skills and allows dynamic modeling of skill development in students (Margaret et al., 2019).

Student modeling is essential in adaptive learning systems, and traditional static student models have limitations in capturing the dynamic nature of learning. To overcome these limitations, DBNs have been proposed as a probabilistic graphical model for student modeling. DBNs represent the student’s knowledge and skills as hidden states that evolve over time, and observations of the student’s interactions with the learning environment are used to update the model and make predictions about future performance. The methodology for constructing DBNs involves defining hidden states, specifying transitions, and estimating parameters from the data. Various tasks such as predicting performance, diagnosing knowledge gaps, and generating personalized recommendations for remedial actions can be performed using constructed DBNs (Käser et al., 2017).

Existing approaches and interventions, such as traditional instructional methods, assistive technologies, and inclusive design principles, are reviewed. The authors also explore the potential benefits of gamification techniques for learning, including increased engagement, motivation, and learning outcomes, specifically in the context of supporting children with LDs. Furthermore, the review of the literature explores existing frameworks for creating inclusive and accessible learning environments for students with dyscalculia, including Universal Design for Learning (UDL) (Shaban & Pearson, 2019), instructional design principles, and accessibility guidelines. The need for a comprehensive framework that integrates inclusive design, instructional design, and gamification techniques to support the unique needs of children with LDs is emphasized, highlighting the gap in the current literature. The literature review serves as a foundation for the authors’ proposed learning design framework, which aims to address this gap and provide a holistic approach to designing inclusive and engaging learning environments for this population (Shaban & Pearson, 2019).

Gamification has also emerged as a promising strategy to enhance participation in science education. Their study demonstrates that tailored game elements, such as adjustable difficulty levels and personalized feedback, can significantly increase the intrinsic motivation and participation of learners. This aligns with broader educational trends that advocate for learner-centered approaches that adapt to individual needs and preferences (Zourmpakis et al., 2023).

The design and evaluation of the computer-based training program Calcularis (Mislevy, 2000) to improve numerical cognition provides information about the design and evaluation of a computer-based training program aimed at enhancing numerical cognition. It highlights the potential of technology-based interventions, such as Calcularis, in supporting individuals with learning difficulties or disabilities to improve their numerical skills. More research in this area is warranted to validate the effectiveness of Calcularis and other similar interventions and explore their broader applications in educational settings (Mislevy, 2000).

Creating an adaptive learning environment that personalizes the learning experience is a crucial goal in modern education. Using data-driven approaches, it is possible to model mathematical skill topologies and optimize the learning process by providing an optimal level of cognitive stimulation. This can involve using scaffolding techniques, segmenting content, and providing customized feedback to meet the unique needs of each learner (Sweller et al., 2019).

Furthermore, predictive analytics can be used to forecast knowledge acquisition and short- and long-term performance for students with dyscalculia, helping in the development of targeted interventions (Siemens, 2013). Introducing a specific design for numerical stimuli, such as visual aids and interactive simulations, can enhance various representations of mathematical concepts. This facilitates understanding through gamified learning approaches that promote engagement and motivation. Individual responsiveness plays a crucial role in helping learners with dyscalculia improve their mathematical skills. Evaluating the impact of interventions is essential to effectively tailor support for individuals with dyscalculia. By collaborating with stakeholders and using their expertise, a comprehensive support system can be established to address the unique needs of learners with disabilities and optimize their mathematical learning experiences.

Solid mathematical skills are crucial for academic success, daily life, and the general well-being of a child, as weaknesses in this area can significantly impact school performance, career choices, and emotional health (El-Sabagh, 2021).

In the context of dyscalculia, a learning disability that affects mathematical ability, the importance of addressing these challenges through effective interventions becomes even more critical. The insights gained from this review of the literature highlight the importance of adaptive technology and personalized feedback in the intervention of dyscalculia. By using advanced models such as Dynamic Bayesian Networks (DBNs) for knowledge tracing, the EDSense system can help customize interventions to meet the specific needs of students with dyscalculia. This approach allows educators and researchers to analyze assessment data more effectively, integrating detailed and accurate information on student performance in mathematical tasks to better inform personalized strategies (Jadhav et al., 2023).

The literature on dyscalculia and mathematical learning difficulties highlights several key aspects, including core deficits in number sense, difficulties in arithmetic operations, challenges with time and money management, and memory-related difficulties. Existing research explores various interventions, including adaptive learning technologies, Bayesian networks for skill modeling, gamification, and inclusive design frameworks. Additionally, tools such as Smartick and Calcularis have demonstrated the potential to provide personalized assessments and improve numerical cognition.

Several studies have examined adaptive learning approaches, emphasizing the role of Bayesian networks and DBNs in modeling skill topologies. These probabilistic models have been utilized to analyze knowledge structures, predict student performance, and recommend personalized learning paths. Furthermore, gamification techniques and Universal Design for Learning (UDL) frameworks have been explored to enhance engagement and accessibility in mathematical learning for children with dyscalculia.

Despite these advances, significant gaps persist in the literature, particularly in the validation and optimization of adaptive learning solutions for children with dyscalculia. Addressing these gaps is essential to enhance the effectiveness and accessibility of technology-driven interventions.

- Limited empirical validation—While theoretical models and pilot studies suggest promising outcomes, large-scale empirical studies that validate the effectiveness of adaptive learning systems and Bayesian models in classroom settings in the real world are lacking.
- Longitudinal studies—There is a lack of longitudinal research tracking the long-term effectiveness of technological and pedagogical interventions in improving mathematical cognition, making it difficult to assess sustained impact.
- Personalized learning strategies—More studies are needed to refine how personalized feedback mechanisms can be optimized for different cognitive profiles of children with dyscalculia, ensuring tailored and effective learning experiences.
- Integration of multiple interventions—Research often focuses on isolated interventions (e.g., gamification, adaptive learning, and assistive technology) without examining their combined effects on children with dyscalculia, which could provide a more comprehensive support system.
- Equity and accessibility—The role of socioeconomic factors in accessing advanced educational technologies remains underexplored, limiting the broader applicability of existing interventions and potentially widening the educational gap.

Future research should prioritize developing comprehensive frameworks that incorporate various instructional strategies while ensuring accessibility and equity in mathematical education for all learners.

## 3. Methodology

**Figure 1:**
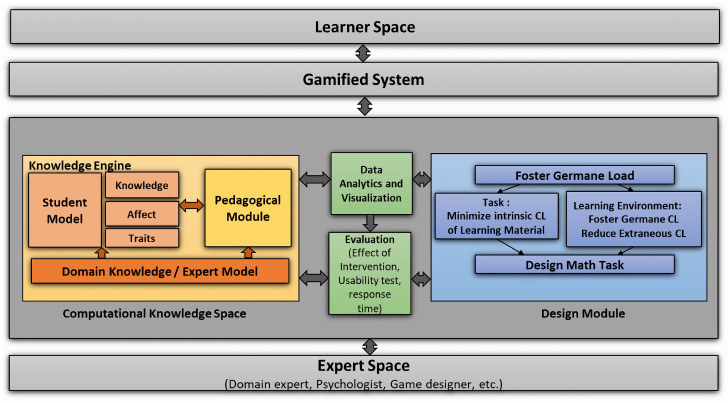
EDSense tool design: Proposed framework.

This research proposes a framework for gamified self-directed learning, designed to model and predict student knowledge using key components of an intelligent tutoring system, as illustrated in Figure 1. The framework consists of several interconnected components, each of which plays a vital role in enhancing the learning experience. The details of these components are presented in Table 1.

To achieve gamified self-directed learning, it is essential to establish a clear hierarchy and the relationships between various mathematical skills within a learning domain. The proposed approach enhances the representational power of the Student Model by using DBNs, enabling the representation of complex skill topologies. The objective is to predict knowledge acquisition through gamified self-directed learning while also evaluating the effectiveness of remedial measures and interventions for children with dyscalculia, specifically focusing on improving their mathematical skills.

Adaptive learning should utilize the unobservable and dynamic nature of a learner’s level of knowledge to support cognitive development. To facilitate efficient learning, it is imperative to incorporate the knowledge structure of the learning elements and establish logical learning paths. Adaptive learning presents significant challenges in achieving its objectives. Three major technical challenges include the following:

- Unobservable and evolving knowledge level—The level of knowledge of learners cannot be directly observed and is a continuous, evolving construct. Therefore, it is challenging to determine the appropriate learning path for the learner.
- Incorporating knowledge structure—Creating logical learning paths requires the incorporation of the knowledge structure of the learning items. The knowledge structure is an intricate network of concepts that makes it difficult to design appropriate learning paths, particularly for complex topics.
- Maximizing overall learning performance—A good recommendation of the learning path should maximize the overall gain throughout the learning trajectory, not just in a particular step. This requires careful consideration of the interconnection between learning objectives, which can be challenging to achieve due to the complexity of many learning environments.

### 3.1. Computational Knowledge Space

As shown in Figure 1, the computational knowledge space comprises a domain knowledge/expert model that stores the knowledge structure using mathematical skill topologies and is utilized by the Student Model. The Domain Model offers a comprehensive and structured representation of the underlying mathematical concepts and skills, making it easier to organize and retrieve them. The Student Model, on the other hand, captures and maintains information about the student’s current knowledge and skills, and uses this information to provide personalized recommendations for learning and practice. The pedagogical module plays a crucial role in the computational knowledge space by selecting appropriate mathematical tasks for the students based on their performance and learning goals. Using these diverse models within the computational knowledge space, educators and learners can effectively and efficiently navigate the intricate landscape of mathematics education, optimizing learning outcomes and engagement.

### 3.2. Student Model

The Student Model is an integral component of the Knowledge Engine shown in Figure 1. It functions as a mathematical construct that embodies a student’s traits, learning capabilities, and performance levels. This framework employs machine learning algorithms to predict both short-term and long-term levels of knowledge and performance. Furthermore, it can also capture and predict various student characteristics, such as group information and long-term performance trends.

The knowledge structure represents a set of mathematical skills in a hierarchical structure, where skills are interconnected through directed connections indicating prerequisites. The Bayesian network is used to calculate the probability of mastering a skill based on the probability of mastering its prerequisite skills. The knowledge structure is initialized with all probabilities set to 0.5, following the principle of maximum entropy. This is carried out because there is no prior knowledge about learners’ mathematical proficiency at the start of training. The DBNs have a memory of five, meaning posteriors are calculated on the basis of the last five time steps.

The design of the knowledge structure was informed by experts’ knowledge of the domain and is based on the assumption that to master a skill, a learner must first master all of its precursor skills. Each skill is assigned to a single task, and the resulting Student Model contains 38 different skills ordered hierarchically according to number ranges (0–10, 0–100, and 0–1000). The model is related to partial-order knowledge structures, which also model dependencies between skills using conditional probabilities.

The proposed approach utilizes DBNs to model the student’s learning process, incorporating relevant domain knowledge. The developed control algorithm is decision-based and facilitates optimization of the learning process through targeted cognitive stimulation. This enables the system to provide personalized feedback and guidance to the student, improving their overall learning outcomes.

A Directed Acyclic Graph (DAG) is a fundamental structure in probabilistic graphical models, consisting of nodes connected by directed edges without any cycles. This acyclic nature ensures a unidirectional flow of influence, making DAGs particularly suitable for modeling causal relationships among variables. In probabilistic modeling, DAGs serve as the backbone for Bayesian networks and Dynamic Bayesian Networks, enabling the representation of complex joint probability distributions through simpler local conditional dependencies.

Recent advancements have focused on improving the learning and inference of DAG structures from data. For example, Annadani et al. (2023) introduced ProDAG, a variational inference framework that projects continuous distributions on the DAG space, facilitating efficient and accurate structure learning. Similarly, Thompson et al. (2024) proposed BayesDAG, a scalable Bayesian causal discovery method that combines stochastic gradient Markov chain Monte Carlo with variational inference to sample DAGs directly from the posterior distribution.

In the realm of student modeling, DAGs are instrumental in capturing the evolving relationships between a learner’s knowledge, skills, and performance over time. By structuring these dependencies, educators and researchers can develop interpretable models that track and predict student learning trajectories, thus informing personalized instructional strategies.

The proposed method creates a DAG for the addition, subtraction, and multiplication of 2-digit numbers. The Student Model is developed using various metrics such as response time, the correctness of the answer, the specific wrong answer (e.g., addition-like multiplication, no carry, etc.), hint usage, and history of attempts to capture the student’s performance and level of mastery. For example, the Student Model can be developed for addition and subtraction within the ranges of 0–10, 0–100, and 0–1000, respectively. The model also considers common mistakes made by dyscalculic children and tailors the feedback accordingly. Furthermore, the Student Model is developed for the multiplication of 2-digit numbers, which is a complex topic for children with dyscalculia. The DAG is utilized to represent the different concepts and sub-skills related to multiplication and to track the student’s progress through the learning process. The Student Model takes into account the student’s performance across different states and diverse subgroups to offer personalized feedback and support. Using these techniques and tools, a supportive and effective learning environment is created for dyscalculic children, ultimately leading to improved learning outcomes and participation.

**Figure 2:**
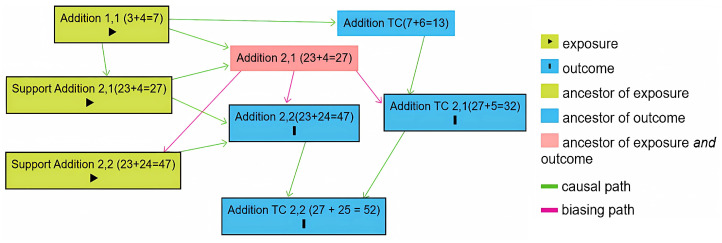
DAG for addition in the number range 0–100 (DAGitty tool).

The proposed approach uses a DBN to model mathematical knowledge. The network is represented as a DAG that captures the relationships among different mathematical skills. The skills are interconnected based on their interdependencies, meaning that two skills, precursor skill A and successor skill B, will have a direct connection if having skill A is a necessary condition for having skill B. Since skills cannot be observed directly, the system infers them by presenting specific tasks and assessing user responses, which are then assigned to the corresponding skills. Figure 2 demonstrates the DAG for addition, created using DAGitty, a web-based tool for building, editing, and analyzing causal diagrams. This tool can also be used to design DAGs for other arithmetic operations (Textor et al., 2011).

The skills in the domain of arithmetic operations are organized according to their level of difficulty. The illustration in Figure 2 shows the skills for addition from 0 to 100. The difficulty of a task depends on factors such as the magnitude of the numbers involved, the complexity, and the method of solving it. For example, solving 23 + 24 = 47 (Addition 2,2) is more challenging than computing 3 + 4 = 7 (Addition 1,1) since the former involves larger numbers. Moreover, a task involving bridging to 10, such as 27 + 5 = 32 (Addition 2,1 with bridging to 10), is more complex than a task without any bridging. Furthermore, for the modeling task 23 + 4 = 27 (Addition 2,1 with support), it is easier to solve the problem mentally (Addition 2,1). The “Subtraction” skills follow the same structure as the “Addition” skills, which means the Subtraction knowledge structure can be derived by substituting Addition with Subtraction in Figure 2. Each skill exists in one of two states: learned or unlearned.

In the proposed approach, a DBN is used to compute the probability of a skill being in the learned state. The Bayesian knowledge tracing method is used to predict a student’s knowledge of a particular skill, taking into account whether they answered correctly, incorrectly, or made a mistake (Slip). The probability of a skill being learned or not being learned is initialized to 0.5 and is updated after each trial based on the student’s performance. The probability of a skill can also be influenced by solving tasks associated with precursor or successor skills. Let us denote *K_t* to represent the knowledge state variable at time *t*, where *K_t* = 1 denotes that the student knows the concept, and *K_t* = 0 denotes that the student does not.

*H_t* represents the hints received at time *t*.

*A_t* represents the attempts made at time *t*.

*C_t* represents the number of correct answers at time *t*.

*W_t* represents the number of wrong answers at time *t*.

*SW_t* represents the number of specific wrong answers at time *t*.

The Sum-Product algorithm (Sánchez-Cauce et al., 2022) is used to calculate the posterior probability of *K_t* = 1 given the observations at time *t*. We will apply the given conditional probabilities and the initial value *P*(*K_t* = 1|*H*_0, *A*_0, *C*_0, *W*_0, *SW*_0) = 0.5.

Given the following conditional probabilities:

$$\begin{array}{c} P(K\_t=1|K_{\left(t-1\right)}=1,H\_t,\;A\_t,\;C\_t,\;W\_t,\;SW\_t)=0.9\\ P(K\_t=1|K_{\left(t-1\right)}=0,H\_t,\;A\_t,\;C\_t,\;W\_t,\;SW\_t)=0.1 \end{array}$$

Step 1. Initialization

(1) P(K_0=1|H_0,A_0,C_0,W_0,SW_0)=0.5

Step 2. Forward Pass

(2) $$\begin{array}{cc} M_{K_{\left(t-1\right)},t} & =P(K\_t=1|K_{\left(t-1\right)}=1,H\_t,A\_t,C\_t,W\_t,SW\_t)\cdot \\ & \;P(K_{\left(t-1\right)}=1|H\_t,A\_t,C\_t,W\_t,SW\_t)+\\ & \;P(K\_t=1|K_{\left(t-1\right)}=0,H\_t,A\_t,C\_t,W\_t,SW\_t)\cdot \\ & \;P(K_{\left(t-1\right)}=0|H\_t,A\_t,C\_t,W\_t,SW\_t) \end{array}$$

(3) $$\begin{array}{c} \;\begin{array}{c} M_{K_{\left(t-1\right)},\;t}=0.9\cdot P\left(K_{\left(t-1\right)}=1|H\_t,\;A\_t,\;C\_t,\;W\_t,\;SW\_t\right)\\ +0.1\cdot P(K_{\left(t-1\right)}=0|H\_t,\;A\_t,\;C\_t,\;W\_t,\;SW\_t) \end{array} \end{array}$$

Step 3. Calculate the Posterior Probability

(4) $$\begin{array}{cc} P(K_{t}=1|H\_t,A\_t,C\_t,W\_t,SW\_t) & =\frac{M_{1,t}P\left(K_{\left(t-1\right)}=1|H\_t,A\_t,C\_t,W\_t,SW\_t\right)}{\sum_{K_{\left(t-1\right)}}M_{K_{\left(t-1\right)},t}P\left(K_{\left(t-1\right)}|H\_t,A\_t,C\_t,W\_t,SW\_t\right)}\\ \; & \;+\frac{M_{0,t}P\left(K_{\left(t-1\right)}=0|H\_t,A\_t,C\_t,W\_t,SW\_t\right)}{\sum_{K_{\left(t-1\right)}}M_{K_{\left(t-1\right)},t}P\left(K_{\left(t-1\right)}|H\_t,A\_t,C\_t,W\_t,SW\_t\right)} \end{array}$$

Step 4. Final Result

The final result will be the posterior probability obtained from the above calculation.

(5) P(K_t=1∣H_t,A_t,C_t,W_t,SW_t)

The Sum-Product algorithm is used to propagate probabilities using conditional probabilities and calculate the posterior probability of K_t=1 given the observations at time *t*. It can be extended to calculate the posterior probabilities for further time steps (e.g., t=2,  t=3, etc.) using the previously computed probabilities from Equation (5). The values of H_t,  A_t,  C_t,  W_t, and SW_t would depend on the specific problem or context.

The Sum-Product algorithm is applied to compute the posterior probability of a student knowing a concept (K_t=1) at time *t*, given a set of observations. The process begins with an initial probability, as per Equation (1), P(K_0=1|H_0,A_0,C_0,W_0,SW_0)=0.5. The algorithm then performs a forward pass, represented by Equations (2) and (3), where it calculates an intermediate message $M_{K_{\left(t-1\right)},t}$ using the given conditional probabilities. This intermediate message incorporates the probabilities that the student either knows or does not know the concept at the previous time step, and it is adjusted by the conditional probabilities of transitioning to the current state. The final step is Equation (4); it involves calculating the posterior probability P(K_t=1|H_t,A_t,C_t,W_t,SW_t) by normalizing the product of the intermediate message and the prior probability. This iterative process allows the algorithm to dynamically update the student’s knowledge state using new observations at each time step. The result shown in Equation (4) is a posterior probability that reflects the student’s updated knowledge state, which can be extended for subsequent time steps by repeating the process with new data.

Inference in this challenges the Student Model to update the posterior probability of the knowledge state variable at each time step based on observed hints, attempts, correct answers, wrong answers, and specific wrong answers. It is used to make predictions or decisions about the student’s knowledge state at each time step or in future time steps.

In this research, the Student Model is utilized by the pedagogical module to make teaching decisions; the module is responsible for optimizing the sequence of tasks presented to the student and deciding when to stop teaching a particular skill based on the current state of the user provided by the Student Model.

The proposed system for dyscalculic children operates based on probability distributions, analyzing multiple observations to estimate the likelihood of unknown variables. These unknown variables include the following:

- Student knowledge—Determining whether a student has acquired specific skills at a given time (*t*).
- Affective state—Assessing the student’s engagement level, such as attentiveness, boredom, or lack of concentration. Recognizing these states enables precise adaptation of training to individual needs.
- Student traits—Factors such as learning behavior and learner type significantly influence learning outcomes. For example, a student trait may indicate whether the student has a learning disability, which affects how the system tailors instruction.

To ensure optimal learning experiences, the domain knowledge/expert model (Figure 1) plays a crucial role in accurately representing skills and learning behaviors. Furthermore, the Analytics and Visualization Engine (Figure 1) uses machine learning to analyze data and provide visual representations, improving the adaptability and effectiveness of the system.

### 3.3. Pedagogical Module

In the pedagogical module, a controller uses posterior probabilities provided by the Student Model to determine the next task. There are three options to choose from: (a) continuing the current skill, (b) going back to a precursor skill, and (c) moving forward to a successor skill. A rule-based approach is used to select the best course of action when multiple precursor and successor skills are available.

Algorithm 1 is proposed as a strategy for creating a personalized training path for a student based on their current knowledge state. It works by using skill-topology/DAG, which is a set of interconnected skills arranged in a hierarchy, to guide the student through different levels of difficulty.
**Algorithm 1** EDsense-Pedagogical module control algorithm  **PLT**—Posterior probability Lower Threshold  **PUT**—Posterior probability Upper Threshold  PLT←0  PUT←1  n_samples←0  current_task←first_task  **while** current_task≤last_task **do**   current_task←play_task(current_task)▷ Play the current task and update it;   posterior_probs←student_model.posterior_probs(current_task.skills)▷ Fetch posterior probabilities   **if** PLT≤posterior_probs≤PUT **then**    Training_level←current_task    current_task←add_successor(current_task)▷ Move to the successor task   **else if**
posterior_probs>PUT
**then**    Training_level←precursor_task    PUT←PUT−Some_amount/n_samples▷ Decrease the upper threshold   **else**    Training_level←successor_task    PLT←PLT+Some_amount/n_samples▷ Increase the lower threshold   **end if**   n_samples←n_samples+1▷ Update the number of samples   train(current_task, Training_level)▷ Perform training at the optimal level  **end while**

Algorithm 1 starts at the lowest level of difficulty and uses a function called *play_task()* to provide the student with a task to complete. The student then uses their knowledge to complete the task, and the student_model computes the probability that the student has mastered the skill required for the task.

If the model determines that the student has not yet mastered the skill, the algorithm moves the student back to a lower level of difficulty to remediate any typical errors they may have made. The skill that corresponds to the lowest probability of mastery is displayed to the student as the remediation skill.

Once the student has completed the remediation skill, the algorithm moves them forward to the next level of difficulty, and the process repeats. The algorithm continues until the student has completed all the skills in the skill topology.

The *play_task()* function gives appropriate tasks based on the student’s skill level, and the student_model computes the probabilities of skill mastery. The train() function updates the knowledge state of the model using the task and the current skill level.

In the context of dyscalculic learners, understanding and supporting their mathematical learning process is of significant importance. The DAG used in our research provides insight into the specific mathematical concepts and operations that pose challenges to dyscalculic learners. This DAG representation allows us to identify critical points of difficulty and develop targeted interventions to support their learning.

In this research, we have selected the Sum-Product algorithm for the inference task. This algorithm is well-suited for acyclic graphs like the one used in our research, as it provides exact results and ensures the precision of inferred probabilities (Sánchez-Cauce et al., 2022). Although other approximate algorithms exist, such as Pearl’s belief propagation, the precision and exactness offered by the Sum-Product algorithm make it the preferred choice. By leveraging the insights gained from the DAG and employing the Sum-Product algorithm, we can effectively understand and address the challenges faced by dyscalculic learners, ultimately improving their mathematical learning experiences. This algorithm dynamically adjusts the difficulty level of tasks based on student performance, continuously assessing the posterior probabilities of task-related skills. By tailoring the level of training to the precursor, current, or successor tasks, the algorithm ensures that each dyscalculic student receives appropriate, challenging, and supportive instruction.

This personalized and adaptive approach not only identifies and addresses individual learning gaps but also fosters continuous improvement and engagement. Consequently, this method offers a significant advancement over traditional techniques, providing a robust framework to enhance the educational outcomes of students with dyscalculia.

### 3.4. Design Module

The Design Module is used to help dyscalculic children learn effectively, as shown in Figure 1; it needs to consider how they process information, how to design learning materials and environments, and how to make learning fun. We reviewed the research and guidelines to design a user-friendly game that minimizes cognitive overload for children.

To optimize learning outcomes for dyscalculic children, it is essential to consider various factors related to cognitive load theory, multimedia learning design, gamification design frameworks, and human–computer interaction (HCI) concepts. A comprehensive review and evaluation of the relevant research, theory, and guidelines have been used to design a gamified application for children with LDs. This aims to maximize usability and minimize cognitive overload by reducing extraneous load, managing intrinsic load, and increasing germane load. The design is based on three categories: a. user guidelines, b. learning materials guidelines, and c. learning environment guidelines. EDSense is designed to foster germane cognitive load by allowing the child to control the application through navigation and support elements (Norman, 2013).

EDSense includes a visual password, a narration option, and constructive audio feedback for correct and incorrect responses, as well as specific wrong answers that can be given by children with dyscalculia. The learning environment is designed to decrease extraneous cognitive load through consistency, minimalist and aesthetic design, appropriate help, error messages, and cognitive navigation based on the child’s performance. The prototype was evaluated by a set of expert reviewers through heuristic inspection and user testing with children. Feedback obtained from the evaluation has been used to improve the final application. Table 2 outlines the primary features and functionalities integrated into the EDSense Dyscalculia Assessment Tool. The tool combines adaptive learning algorithms, interactive design, and real-time analytics to support personalized assessment and progress monitoring for learners with mathematical learning difficulties.

## 4. Materials and Methods

### 4.1. Selection Criteria and Participants

**Figure 3:**
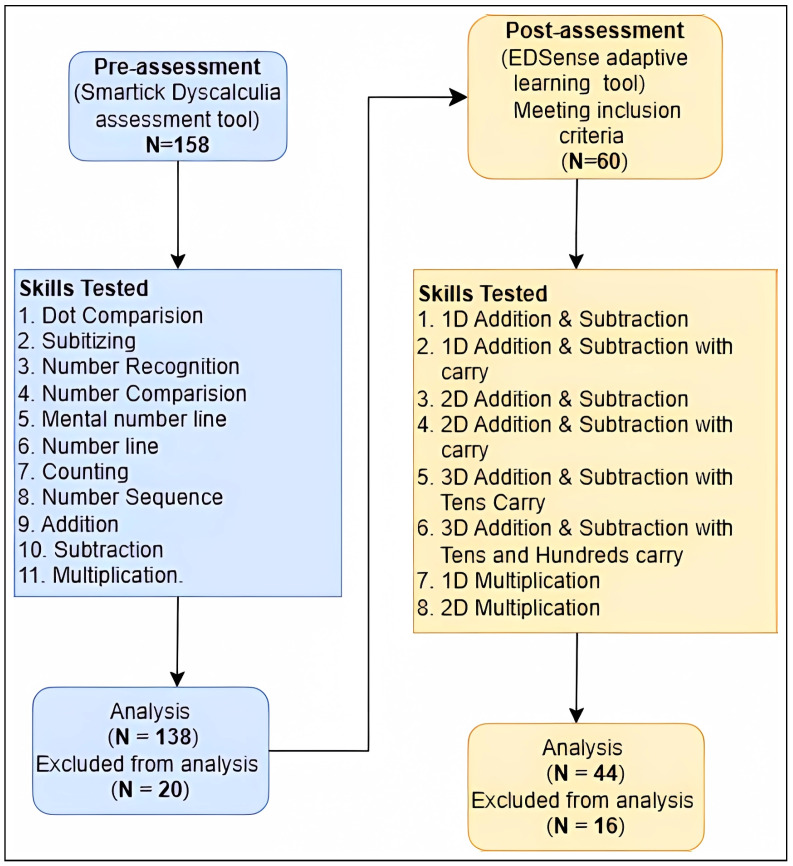
The overview of the research design and sampling.

As shown in Figure 3, the research involved *N* = 158 students, aged 6 to 10 years, identified by their teachers as having low mathematics performance. The primary criterion for selection was a performance level below 50%, as determined by the teachers, using convenience sampling. These students were assessed using the Smartick Dyscalculia Assessment Tool, an audio-visual web-based test administered on PCs. This initial assessment revealed that students’ performance was significantly influenced by their proficiency in using the mouse interface. Inexperienced students faced challenges related to precise cursor control, accurate button/item selection, and navigation within the test interface. Factors such as age, previous mouse use experience, and fine motor skills emerged as potential contributors to these difficulties.

The sample selection for this research was conducted using purposive sampling, a non-probability sampling method where participants were deliberately chosen based on their pre-test results. This approach ensures that the sample aligns with the research objectives by focusing on individuals who meet specific criteria relevant to the research objective, such as performance levels on the pre-test. A total of *N* = 44 participants were required, based on a priori power analysis, to detect a moderate effect size ($d_{z}=0.56$) with a significance level of (α=0.05) and a desired power of (1−β=0.95). The noncentrality parameter (δ=3.7146) and the critical t-value (t=2.0167) indicate that the research is well powered, with an actual power of 0.9526, slightly exceeding the target.

Although purposive sampling is effective in ensuring that the selected participants are directly relevant to the research, it may introduce selection bias and limit the generalizability of the findings to broader populations. However, this method is appropriate for the research design, as the intent is to analyze outcomes within a specific group defined by their pre-test performance. To address potential limitations, efforts were made to clearly define and document the selection criteria to ensure transparency and consistency in the sampling process.

Focusing specifically on a subset of this group, *N* = 44 students of a primary school in Mumbai, India, who scored below the 40th percentile in the initial assessment during their 4th Grade, were selected for further testing. This group consisted of (*N* = 17) males and (*N* = 27) females, with a mean age of 9.12 years (*SD* = 0.86). These students were chosen based on their particularly low performance to gain a deeper understanding of their learning difficulties and to provide targeted interventions.

### 4.2. Tools and Procedures

The primary tool used for the initial assessment was the Smartick Dyscalculia Assessment Tool. This tool was selected for its comprehensive evaluation of mathematical abilities through an engaging audio-visual format. The Smartick tool provided initial information on the student’s mathematical proficiency and highlighted significant challenges related to the use of the mouse interface, which impacted test performance.

Recognizing the need for a more tailored approach to accommodate the specific challenges identified in the initial assessment, our research introduced the EDSense web-based tool for subsequent evaluations. EDSense was specifically designed to meet the unique needs of dyscalculic learners. This tool addressed the difficulties observed with the Smartick test by providing a more adaptive and user-friendly interface, thereby enhancing the accuracy and reliability of the assessments.

To ensure a controlled and conducive testing environment, the EDSense assessments were conducted in a primary school in Mumbai. The testing environment was carefully controlled to minimize external influences, ensuring the reliability of the results. The duration of each assessment was customized, ranging from 30 to 120 min, to accommodate the diverse learning needs within the specified age group. This flexible approach was adopted to ensure that each student’s mathematical abilities were thoroughly evaluated, considering their pace and proficiency.

### 4.3. Analysis Plan

The analysis plan involved evaluating the mathematical ability of 44 primary school students using the EDSense assessment tool, which tested various mathematical skills such as Addition, Subtraction, and Multiplication. The speed and accuracy of responses were analysed across different operations and difficulty levels. Welch’s ANOVA (Analysis of Variance) was used to determine whether response time differences between difficulty levels were statistically significant, considering the violation of the assumption of homogeneity of variances. This method ensures a more reliable assessment when the assumption of equal variances is not met. Error analysis was performed to detect specific learning difficulties, such as dyscalculia, by identifying patterns such as operation confusion, misplacement of numbers, and misconceptions involving zero. The results were then mapped and visualized to better understand individual student performance and learning challenges. The results are categorized and presented in key dimensions, including participant performance, arithmetic operation trends, variability between levels of complexity, and individual learning difficulties, offering valuable insight into the various cognitive profiles and needs of young learners.

## 5. Results and Discussions

The primary objective of this research was to evaluate the mathematical performance of students aged 6 to 10 years by implementing and evaluating the EDSense tool. The results are categorized into key areas: participant performance, speed and accuracy analysis, response time evaluation, dyscalculia-related challenges, and student performance trends. In this research, the primary objective revolved around a comprehensive investigation of the mathematical performance of students aged 6 to 10 years, specifically encompassing the first to fourth standard levels. The focal point of the investigation was the utilization and evaluation of the EDSense tool. This research was carried out within the educational setting of a Mumbai-based primary school, where a targeted group of *N* = 44 students from the fourth standard were selected as participants.

### 5.1. Participant Performance Overview

**Figure 4:**
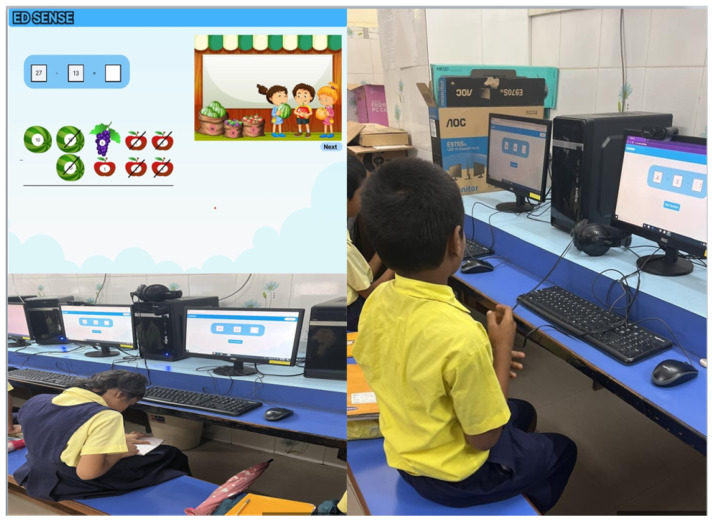
EDSense tool and assessment environment.

This research was carried out in a Mumbai-based primary school with a sample of 44 students of the fourth standard. The evaluation process was structured to accommodate the unique learning needs of each student, with test durations ranging from 30 to 120 min. Despite minor technical difficulties with the mouse interface, collaborative efforts between teaching staff and student assistants ensured smooth administration of the research. The experiment setup is shown in Figure 4.

To ensure smooth and effective administration of the research methodology, meticulous planning was executed. However, various challenges emerged during the process. Collaborative efforts were initiated, involving both the school’s teaching staff and a team of four student assistants. These collaborations served to provide invaluable assistance during the audio-visual test, despite some participants facing difficulties with the mouse interface, as they were not accustomed to operating a PC. Although present, these challenges did not hinder the overall testing process, highlighting the importance of the collaborative approach in capturing accurate data.

This research sheds light on the methodology, assistance, and duration aspects of the research conducted at the Mumbai-based primary school. By focusing on the implementation of the EDSense tool and its impact on mathematics learning, the research seeks to contribute valuable insights to the realm of educational practices and strategies targeted at fostering mathematical proficiency among young learners.

### 5.2. Speed and Accuracy Analysis

Table 3 provides statistics for speed and accuracy measures in the Smartick pre-assessment of various skills. The variance and standard deviation values for each skill in terms of both speed and accuracy provide insight into the variability and consistency of performance. For instance, in the “Dot Comparison” skill, higher speed variance is accompanied by lower accuracy variance, suggesting a potential trade-off between speed and accuracy in this specific task. Similar patterns can be observed across different skills, providing a detailed overview of the distribution of performance metrics in the pre-assessment.

The findings presented in Table 3 significantly enrich our understanding of the complex interplay between speed and accuracy measures, providing nuanced insights into their relationship within the specific context of the research.

Table 4 presents correlation coefficients and standard errors for the relationship between speed and accuracy measures. The negative correlation coefficients (−0.5176 and −0.5034) indicate an inverse relationship between speed and accuracy variance, as well as speed and accuracy standard deviation. These findings suggest that in the context of the research, there is a tendency for increased speed to be associated with decreased accuracy, and vice versa, while standard errors provide insights into the precision of these correlations.

Taking into account the presence of dyscalculia, a learning disability that affects mathematical skills, this research approaches the study with sensitivity. Dyscalculia can lead to challenges in number recognition, calculation, and problem-solving. Analyzing response times for various mathematical operations should acknowledge potential delays in students with dyscalculia. The research seeks to understand how students with dyscalculia respond to varying complexities, providing information for customized educational approaches and accommodations. This contributes to the promotion of inclusivity in mathematical learning environments.

The analysis of the response times for arithmetic skills reveals different patterns in the “Addition”, “Subtraction”, and “Multiplication” tasks. Furthermore, the data exhibit greater variability, indicating a diverse range of response times between individuals. Subtraction, on the other hand, demonstrates more consistent performance, with lower variance and standard deviation, suggesting a narrower distribution of response times. “Multiplication” falls between “Addition” and “Subtraction” in terms of variability, indicating a moderate range of response times. These insights are valuable in understanding the cognitive demands associated with each arithmetic operation, potentially guiding educational strategies and assessments.

**Figure 5:**
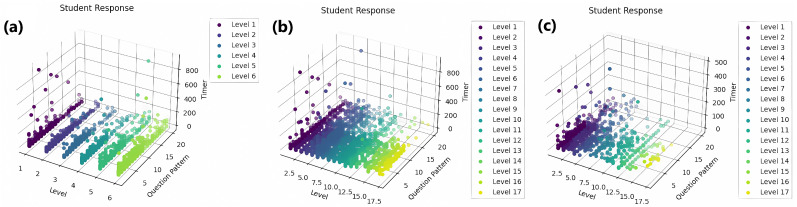
Skill-wise response time analysis (**a**) Multiplication (two-digit) (**b**) Addition within the 0–1000 range (**c**) Subtraction within the 0–1000 range.

The response time analysis is visually depicted through a series of three-dimensional (3D) line graphs in Figure 5a–c, each dedicated to examining response times concerning “Addition”, “Subtraction”, and “Multiplication”, respectively, across 17 distinct levels of mathematical complexity.

### 5.3. Response Time Evaluation

Table 5 presents the response time statistics for the skills assessed in the EDSense evaluation. The variance and standard deviation values for “Addition”, “Subtraction”, and “Multiplication” indicate the degree of variability and consistency in the response times of the participants. For example, the higher variance and standard deviation in the “Addition” skills suggest greater variability and dispersion in response times compared to “Subtraction” and “Multiplication”. The response times for Addition, Subtraction, and Multiplication have mean values of 49.80 min, 44.64 min, and 37.00 min, respectively. These averages reveal the relative baseline response time demands for each skill. These metrics provide valuable information on the temporal aspects of skill performance, helping to understand the dynamics of the cognitive processes associated with each arithmetic operation in the EDSense assessment.

The results of Welch’s ANOVA indicate significant differences in response times across difficulty levels for the three operations: Addition, Subtraction, and Multiplication, as shown in Table 6. For Addition, (F = 11.89, *p* < 0.0001) demonstrate that as the difficulty increases, the response times increase significantly. Similarly, in the Subtraction, a significant effect of difficulty level is observed (F = 14.04, *p* < 0.0001), indicating higher cognitive demand at more complex levels. Multiplication also shows a significant increase in response times across difficulty levels (F = 13.73, *p* < 0.0001), reflecting the growing task complexity with increasing level.

These results suggest that individuals, particularly those with dyscalculia, will experience greater delays as task complexity increases. This pattern emphasizes the need for targeted interventions and support for individuals struggling with basic arithmetic tasks, especially as they progress to more complex mathematical operations.

The tabular presentation in Table 7 summarizes the key statistical characteristics of our dataset, shedding light on the performance and assessment metrics used in this research. The Correct_Answers_Level across all levels from 1 to 1.17, 2 to 2.17, and 3 to 3.6 and the Correct_Answers_Questions 10 attempts per level across all levels demonstrate an average performance level of approximately 2.04 and 5.31, respectively.

Table 7 summarizes key metrics from a sample dataset, focusing on various aspects of participants’ performance. In particular, metrics such as Correct_Answers_Level and Wrong_Answers_Level provide insights into average correctness levels and the prevalence of incorrect responses per level, respectively. The Specific_Wrong_Level metric indicates the average occurrence of specific wrong answers per level, and the Special_Case metric highlights instances of a special case.

Across these metrics, the dataset showcases variability in participant performance, providing a detailed understanding of correctness, errors, and unique occurrences. These findings are a valuable foundation for further analysis and interpretation of the participants’ behavior in the context studied.

Table 8 summarizes the average response times in various stages and skill levels in mathematics. The data indicate that Addition tasks generally require more time, especially at higher levels, suggesting increased cognitive demands. In contrast, Multiplication shows lower response times at initial levels, reflecting ease with basic concepts. Subtraction times exhibit notable variability, with specific peaks indicating particular difficulties. This analysis highlights the evolving complexity of mathematical operations as learners advance, emphasizing the need for targeted support at higher skill levels.

### 5.4. Dyscalculia-Related Challenges

**Figure 6:**
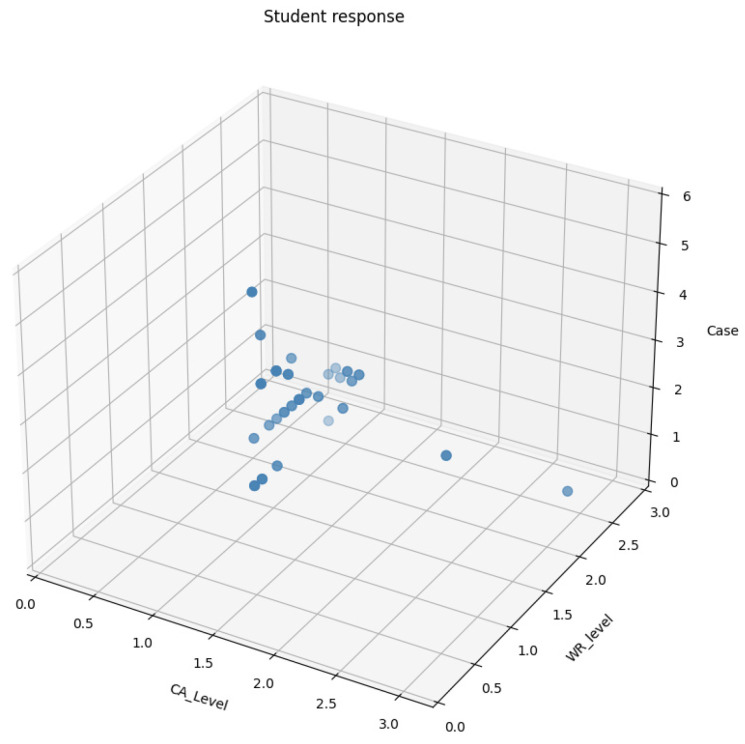
Anomalies and special cases: experimental findings.

In the EDSense research, we investigated five specific cases of dyscalculia, each related to different memory challenges. Some participants had difficulty recalling basic arithmetic facts (semantic memory), and others struggled to understand and use mathematical procedures (procedural memory). Another group faced difficulties with visuospatial memory, meaning that they had difficulty grasping numerical information presented visually, such as column alignment, place value errors, or geometry problems (Cornue, 2018). We carefully examined and visually represented these findings in Figure 6 to highlight unique cases and anomalies within our experiment.

Case 1 (Semantic memory—Operational misattribution): Some participants exhibited a tendency to treat Addition as Multiplication, blurring the lines between these fundamental mathematical operations, e.g., 3 + 5 = 15 or 32 + 7 = 109.

Case 2 (Visuospatial memory)—Another group struggled with multidigit Addition and Subtraction, consistently failing to carry over or borrow digits, resulting in all digits remaining clustered at the bottom of their calculations, e.g., 28 + 14 = 312.

Case 3 (Procedural memory—Disorganized arithmetic strategy)—A separate set of individuals showed a unique challenge, as they approached arithmetic by adding or subtracting digits individually, disregarding the need for a structured digit-by-digit approach, e.g., 92 + 43 = 18.

Case 4 (Procedural memory—Vanishing digits)—In contrast, some participants experienced “vanishing digits” during their calculations, often forgetting or inadvertently omitting certain digits during Addition or Subtraction, e.g., 39 + 25 = 54.

Case 5 (Semantic memory—Zero-effect misconception)—The experiment also revealed a case where participants consistently believed that any mathematical operation involving zero would inevitably produce zero as the outcome, illustrating the profound impact of this misconception on their mathematical reasoning, e.g., 26 + 20 = 40.

### 5.5. Student Performance Trends

**Figure 7:**
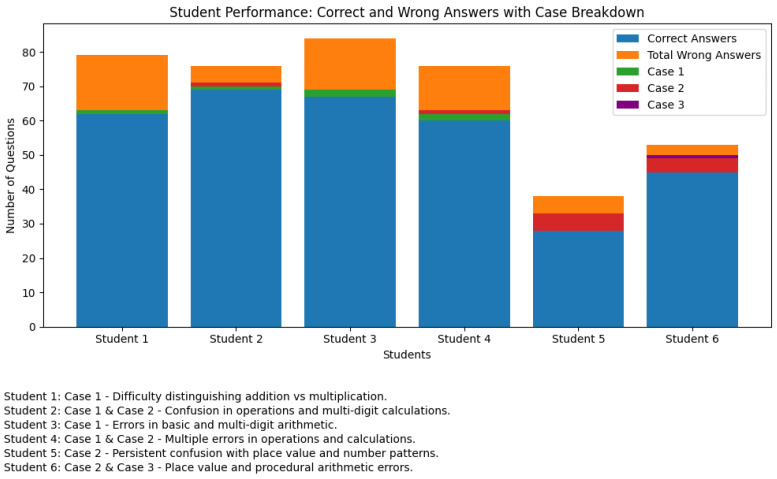
Student performance across different cases.

Figure 7 presents a summary of the performance of the selected students at different levels of arithmetic skills, detailing both correct and incorrect answers. It includes special cases where students encountered specific challenges (like confusion between operations or difficulty with place values), often linked to dyscalculia-related issues. Each row presents a student’s overall performance, the specific arithmetic levels assessed, and detailed remarks explaining errors and their likely causes.

Figure 7 presents a bar graph that illustrates the performance of six students on a test, broken down into correct answers and total wrong answers, with the categorization of wrong answers broken into three distinct cases. The performance of each student is depicted by a set of stacked bars. The first layer of the bars represents the number of correct answers, while the subsequent layers display the distribution of wrong answers using three cases: Case 1, Case 2, and Case 3. Case 1 corresponds to errors related to basic arithmetic operations (e.g., Addition vs. Multiplication), Case 2 captures errors arising from multidigit calculations and place value confusion, and Case 3 highlights procedural or specific arithmetic misunderstandings.

As shown in Figure 7, most of the students performed well, with Student 1 through Student 4 achieving a high number of correct answers. However, even these students exhibited minor mistakes, as indicated by the stacked portions representing incorrect answers and case classifications. Student 5 and Student 6 demonstrated lower overall performance, with a higher frequency of errors, particularly in Case 2 (multidigit calculations and place value issues). In particular, Student 6 also made a Case 3 error, suggesting a more complex misunderstanding in their arithmetic processes.

Figure 7 visually underscores both the overall success of most students and the specific areas where certain students struggled, providing valuable information for targeted educational interventions. This analysis reveals that students with difficulties in arithmetic often exhibit errors that can be attributed to cognitive challenges in various memory systems, such as semantic, visuospatial, and procedural memory. Understanding these cognitive difficulties is essential for developing targeted educational interventions aimed at improving arithmetic skills.

### 5.6. Speed-Accuracy Trade-Off in Mathematics Learning

The negative correlation between speed and accuracy suggests that students who complete tasks faster tend to make more errors, highlighting the need for balanced instructional strategies that encourage both fluency and precision in mathematical tasks (Geary et al., 1992). Additionally, the variability in response time in arithmetic operations indicates different cognitive demands. Greater variability in Addition tasks suggests higher cognitive effort, while Subtraction demonstrates a more consistent pattern. This aligns with EDSense’s research, which shows that students often struggle with Addition more than Subtraction in the early stages of learning.

### 5.7. Dyscalculia Considerations and Individualized Support

The presence of challenges related to dyscalculia reinforces the need for personalized instructional methods. Students facing semantic and procedural memory deficits may benefit from the following:

- Visual aids and step-by-step problem breakdowns.
- Reinforcement exercises for place value understanding.
- Error analysis interventions to correct conceptual misunderstandings.
- Multisensory learning techniques, such as using manipulatives and interactive tools (Butterworth, 2018).
- Adaptive digital learning environments that provide real-time feedback and scaffolding (Geary, 2013).
- Collaborative learning strategies that encourage peer support and discussion to build conceptual clarity (Ramaa, 2002).

Research suggests that early identification and intervention are critical in supporting students with dyscalculia. Integrating assistive technologies and differentiated instructional approaches can improve learning outcomes (Sharma, 2015).

### 5.8. EDSense in Mathematics Learning and Future Research

The EDSense tool provides valuable insights into student performance by tracking response times, accuracy rates, and cognitive challenges. Its adaptive approach allows educators to customize learning experiences to meet individual student needs. Future research should explore its long-term impact on mathematics proficiency and possible modifications to enhance its effectiveness for students with LDs. Further investigations should examine the role of instructional interventions in mitigating speed-accuracy trade-offs, the effectiveness of personalized support strategies for dyscalculia-affected students, and longitudinal studies assessing the sustained impact of digital learning tools like EDSense on mathematical development.

## 6. Conclusions

The challenge of supporting students with dyscalculia, a specific learning difficulty that affects the acquisition of arithmetic skills, has drawn increasing attention in educational research and practice. The reviewed literature highlights the growing role of artificial intelligence (AI), gamified learning environments, and adaptive educational technologies in addressing these challenges in primary education settings.

At the core of this research lies the recognition that students with dyscalculia often face various individualized obstacles that traditional teaching methods cannot overcome. These include difficulties with number sense, spatial reasoning, and working memory. In addition, external factors such as interface familiarity, fine motor skills, and prior digital experience significantly affect the accuracy of dyscalculia diagnoses. These findings underscore the importance of developing tools that are not only diagnostically sound but also accessible and tailored to individual learner profiles.

The Smartick Dyscalculia Assessment Tool was administered to students aged 6 to 10 years with weak mathematical performance to assess dyscalculia. The findings highlight the influence of various factors on the accuracy of the assessment, including mouse interface proficiency, age, prior experience with mouse usage, and fine motor skills. These insights underscore the need for further exploration and targeted interventions to improve mouse proficiency skills in digital assessment environments, ensuring the reliability of dyscalculia evaluations. Addressing these factors is crucial for accurately diagnosing and supporting students with mathematical learning difficulties.

To bridge these gaps, we proposed a technology-driven framework that ensures an adaptive, gamified, and data-driven learning approach. This framework tailors interventions to the specific needs of each student, fostering engagement and enhancing learning outcomes. Building upon this framework, we developed EDSense, an adaptive educational tool designed to support students struggling with mathematical concepts in the Smartick system. Beyond its assessment capabilities, EDSense integrates DBNs within a personalized learning model to predict and assign the next skill based on individual performance, ensuring a customized learning experience.

Despite these advances, the current desktop-based implementation of EDSense presents a limitation. Accessibility remains a concern, especially for younger students or those with limited experience using mouse-driven interfaces, a factor previously identified as influencing assessment outcomes. To address this, future iterations of EDSense can prioritize mobile-friendly and touch-optimized designs that accommodate a wider range of motor skills and user preferences.

This research contributes meaningfully to the field of inclusive education, with dyscalculia intervention at its center, highlighting the need for the following:

- Early, accurate, and multimodal screening tools that consider technological familiarity.
- Gamified frameworks that enhance motivation and cognitive engagement.
- Personalized interventions that adapt in real time to student performance.
- Accessible design choices to ensure that all learners can benefit from digital tools, regardless of physical or technological limitations.

This research contributes to the larger goal of fostering inclusive and personalized learning environments for students with dyscalculia. By identifying and addressing their specific challenges, EDSense not only enhances mathematical proficiency but also plays a crucial role in promoting educational equity. Our proposed framework for adaptive dyscalculia intervention highlights the importance of early detection, data-driven decision-making, and personalized skill refinement. The ongoing research aims to further refine the platform by analyzing how students interact with EDSense’s cues and interventions, ultimately improving its effectiveness as an intelligent tutoring system for mathematical learning difficulties.

## Figures and Tables

**Table 1:** Framework components and descriptions.

Component	Description
Learner Space	The interactive environment where students engage with mathematical tasks and adaptive learning experiences.
Gamified System	Integrates game-based elements to enhance motivation, engagement, and persistence in learning mathematics.
Computational Knowledge Space	Student Model: Captures student knowledge, affective states, and personality traits to personalize learning. Pedagogical Module: Adapts learning paths based on student performance and cognitive needs. Domain Knowledge (Knowledge Engine): Stores structured mathematical concepts and learning hierarchies.
Design Module	Foster Germane Load: Encourages deep learning and meaningful connections. Minimize Intrinsic Load: Simplifies complex concepts to enhance comprehension. Reduce Extraneous Load: Eliminates unnecessary distractions for efficient learning. Design Math Tasks: Creates tailored exercises aligned with cognitive principles.
Data Analysis and Visualization	Processes learning data to provide insights, progress tracking, and personalized feedback.
Evaluation	Assesses system effectiveness, student progress, and learning outcomes using various metrics.
Expert Space	Domain Experts: Ensure accurate and structured mathematical content. Psychologists: Address cognitive and affective aspects of learning. Game Designers: Enhance engagement through game-based strategies.

**Table 2:** Features and functionalities of the EDSense Dyscalculia Assessment Tool.

Aspect	Description
Adaptive Assessment	Dynamically adjusts question difficulty using Bayesian Knowledge Tracing based on learner responses.
Gamified User Interface	Provides an interactive, user-friendly interface and rewards to boost motivation and engagement.
Immediate Feedback	Delivers real-time visual feedback and hints for both correct and incorrect answers.
Mathematical Concept Coverage	Includes Addition, Subtraction, and Multiplication with increasing levels of complexity.
Performance Tracking	Monitors accuracy, response times, and progress over time to support learning diagnostics.
Error Pattern Recognition	Detects and categorizes specific wrong answers to highlight specific learning difficulties.
Progress Reports	Automatically generates analytical progress reports.
MERN Stack Architecture	Built with MongoDB, Express.js, React, and Node.js for scalability, speed, and modularity.

**Table 3:** Speed and Accuracy statistics in Smartick pre-assessment.

Skill Name	Speed		Accuracy	
	Variance	SD	Variance	SD
dotComparison	0.7153	0.8458	1081.714	32.8894
subitizing	0.0286	0.169	1707.3046	41.3195
numberRecognition	0.6335	0.796	1498.5401	38.711
numberComparison	0.1417	0.3764	1723.6636	41.517
mentalNumberLine	0.8028	0.896	882.3046	29.7036
numberLine	0.4742	0.6886	622.0535	24.941
counting	0.4281	0.6543	1519.2679	38.9778
numberSequence	0.4347	0.6593	743.6749	27.2704
addition	0.4045	0.636	1682.6616	41.0203
subtraction	0.714	0.845	1320.8056	36.3429
multiplication	0.5255	0.7249	940.8224	30.6728

**Table 4:** Correlation coefficients and standard errors.

	Correlation Coefficient	Standard Error
speed_and_accuracy_Variance	−0.5176	488.7471
speed_and_accuracy_SD	−0.5034	7.9930

**Table 5:** Response time statistics for EDSense assessment.

Skill Name	Variance	SD
Addition	4552.7869	67.4743
Subtraction	2936.0731	54.1855
Multiplication	3689.9371	60.7448

**Table 6:** Results of Welch’s ANOVA for Response Times Across Different Stages and Operations.

Operation	F-Statistic	*p*-Value	Conclusion
Addition	11.890121	<0.0001	There is a significant difference in response times across levels, indicating that the complexity of the addition task increases as the difficulty level rises.
Subtraction	14.037830	<0.0001	A significant difference in response times is observed across levels. Higher difficulty levels lead to slower response times in subtraction tasks, suggesting a greater cognitive load.
Multiplication	13.733935	<0.0001	Significant differences in response times across levels suggest that task complexity increases with higher levels, impacting the multiplication task’s response times.

**Table 7:** Summary of metrics in the sample dataset.

Metric	Mean Value	Min Value	Max Value	No. of Entries
Correct_Answers_Level	2.04	0.0	3.6	1819
Correct_Answers_Questions	5.31	0.0	10.0	1819
Wrong_Answers_Level	0.82	0.0	3.6	1826
Wrong_Answers_Questions	2.17	0.0	10.0	1819
Specific_Wrong_Level	1.57	1.1	2.6	64
Specific_Wrong_Questions	4.94	1.0	10.0	64
Special_Case	1.96	1.0	5.0	55

**Table 8:** Analysis of average response time across different stages and levels.

Aspect	Observation
General Trends	Response times vary by skill level and stage. Addition times are generally higher than Subtraction and Multiplication, especially at higher levels (Level 6+).
Levels 1–3	Lower response times across all stages, indicating manageable difficulty. Multiplication times are lowest, suggesting ease with basic tasks.
Levels 4–6	Noticeable increase in Addition and Subtraction times, especially at Level 5 and 6, likely due to increased complexity. Multiplication time peaks at Level 6.
Levels 7–10	Addition and Subtraction times increase sharply, suggesting more challenging problems or concepts introduced at these levels.
Levels 11–17	Addition and Subtraction remain high, with Subtraction times peaking at Levels 6 and 17, indicating difficulty or potential fatigue at advanced levels.
Stage-Specific Trends	Addition: Shows a steady increase in response time, peaking around Level 13. Subtraction: High variability with sharp peaks at Levels 3, 6, and 17, suggesting specific difficulties at these points. Multiplication: Low response times at lower levels, reaching a peak at Level 6.
Summary	Initial levels show lower times, indicating introductory complexity. Increasing response times at higher levels suggest a growing cognitive load and problem difficulty.

## Data Availability

The data that support the findings of this study are available upon request. The raw data and processed dataset associated with this research can be accessed by contacting the corresponding author.
